# Integrated electronic skin (e-skin) for harvesting of TENG energy through push–pull ionic electrets and ion-ion hopping mechanism

**DOI:** 10.1038/s41598-021-04555-3

**Published:** 2022-03-09

**Authors:** Ravi Kumar Cheedarala, Jung Il Song

**Affiliations:** The Research Institute of Mechatronics, Department of Mechanical Engineering, Changwon National University, Changwon City, South Korea

**Keywords:** Environmental sciences, Chemistry, Materials science, Nanoscience and technology

## Abstract

The development of highly durable, stretchable, and steady triboelectric nanogenerators (TENGs) is highly desirable to satisfy the tight requirement of energy demand. Here, we presented a novel integrated polymeric membrane that is designed by PEDOT: PSSa-naphthalene sulfonated polyimide (PPNSP)-EMI.BF_4_ Electronic skin (e-skin) for potential TENG applications. The proposed TENG e-skin is fabricated by an interconnected architecture with push–pull ionic electrets that can threshold the transfer of charges through an ion-hopping mechanism for the generation of a higher output voltage (Voc) and currents (Jsc) against an electronegative PTFE film. PPNSP was synthesized from the condensation of naphthalene-tetracarboxylic dianhydride, 2,2′-benzidine sulfonic acid, and 4,4′diaminodiphenyl ether through an addition copolymerization protocol, and PEDOT: PSSa was subsequently deposited using the dip-coating method. Porous networked PPNSP e-skin with continuous ion transport nano-channels is synthesized by introducing simple and strong molecular push–pull interactions via intrinsic ions. In addition, EMI.BF_4_ ionic liquid (IL) is doped inside the PPNSP skin to interexchange ions to enhance the potential window for higher output Voc and Iscs. In this article, we investigated the push–pull dynamic interactions between PPNSP-EMI.BF_4_ e-skin and PTFE and tolerable output performance. The novel PPNSP- EMI.BF_4_ e-skin TENG produced upto 49.1 V and 1.03 µA at 1 Hz, 74 V and 1.45 µA at 2 Hz, 122.3 V and 2.21 µA at 3 Hz and 171 V and 3.6 µA at 4 Hz, and 195 V and 4.43 µA at 5 Hz, respectively. The proposed novel TENG device was shown to be highly flexible, highly durable, commercially viable, and a prospective candidate to produce higher electrical charge outputs at various applied frequencies.

## Introduction

For two decades, fossil fuel reservoirs are gradually decreasing owed to abundant consumption of human society. Alternatively, scientists and engineers are focused to develop clean, and renewable energy techniques which includes piezoelectric, photoelectric, thermoelectric, pyroelectric, electrostatic, and electromagnetic devices to reduce the dependency on fossil fuels^1–5^. They can work as portable energy harvesters to drive low-powered electronic devices and work as self-powered sensors^6,7^. In addition, numerous technologies suffer from issues concerning device design limitations, the development of highly efficient composite films, long processing periods, power control circuits, unsteady production performance, packaging, and shelf-life issues. In recent times, triboelectric nanogenerators (TENGs) have established global commitment for the collecting of viable green energy from ambient resources^8^. TENGs were technologically advanced based on an amalgamation of contact separation electrification and electrostatic stimulation for scavenging attenuated mechanical energy via triboelectric resources^9,10^. The appropriate selection of triboelectric paired materials and their coherent design can upsurge the rate of energy collection and conversion efficiency^11,12^. At regular intermissions of TENG resources with oppositely charged electrets, ions or electrons can be driven to movement through the external load and produce a continuous current^13^.

In recent years, sulfonated polyimide block copolymers (SPIs), and PEDOT: PSSa conducting polymers are being used as the most promising organic polymers that contain regular porous nanochannels and present numerous manufacturing qualities, such as film forming ability with resilience, elasticity, bendability, stretching ability, long shelf life, and electrochemomechanical properties^14–19^. Although several groups have used a series of SPIs as ionomers for high-performance fuel cell applications and actuators but not applied for TENG applications^20,21^. PEDOT: PSSa is a combination of polymer mixture of two conductive ionomers. One of the components in this mixture is made up of sodium polystyrene sulfonate and some of the sulfonyl groups are deprotonated and carry a negative charges^22–24^. Recently, Jang have designed conductivity enhancement experiments by mixing of ionic liquids (ILs) into PEDOT: PSSa conducting polymer for a noteworthy conductivity improvement^25^. The ions exchange between PEDOT: PSSa, and IL can assist PEDOT to decouple from PSSa, and harvest bulk-scaled loosely bound conducting domains^26^. The spontaneous ion exchange followed by nano channeled phase between PEDOT and PSSa chains, with formation of a regular π–π stacked PEDOT cations were intercalated by IL anions, is further sustained by molecular dynamics performed on bulk PEDOT: PSSa models in solution^27–33^. Furthermore, the IL can act as a bridge electrolyte between the core SPI polymer and PEDOT: PSSa electrodes, it can provide a further significant intensification of charge distribution performance and extended durability. In addition, the EMI.BF_4_ is evaluated as a low temperature electrolyte additive, and enhance the cycle stability^34^. Yan et al. have reported porous g-C3N4, and Mxene dual confined FeOOH Quantum Dots for superior energy storage in an EMI.BF_4_ IL for high efficiency supercapacitors^35^. From their exploration, we inspired to use an EMI.BF_4_ for emerging high performance TENG by producing fast ion-hopping rate between cations and hydrophilic NSP.H^+^, and PEDOT: PSSa conduction layers by ion-ion interactions through porous nano channels^36^.

To develop an economically viable, highly endurable, and exchange of interionic ionic electrets between electropositive, and electronegative porous next-worked NSP.H^+^, and PPNSP membranes were used to generated high output voltage and currents through TENG process. Additionally, we presented a simple but ultrafast two-step synthesis including dry casting and drop casting for a high-performance TENG. The ionic electrets networked, hydrogen ion (H^+^) rich naphthalene sulfonated polyimide (NSP.H^+^) ionic membrane, polyethylenedioxythiophene (PEDOT): polystyrene sulfonate (PSSa) as a conducting electrode layer, and 1-ethyl-3-methylimidazolium tetra fluoroborate [EMI.BF_4_] ionic liquid (IL) as the mobile electrolyte^37^. Molecular-level region-specific interaction of cations and anions in IL with hydrophilic-hydrophobic co-blocks of NSP mediocre is utilized for building a self-assembled ionic networked polymer with uninterrupted and intersected ion transport nano channels for high-performance TENG^38^.

**Figure 1:**
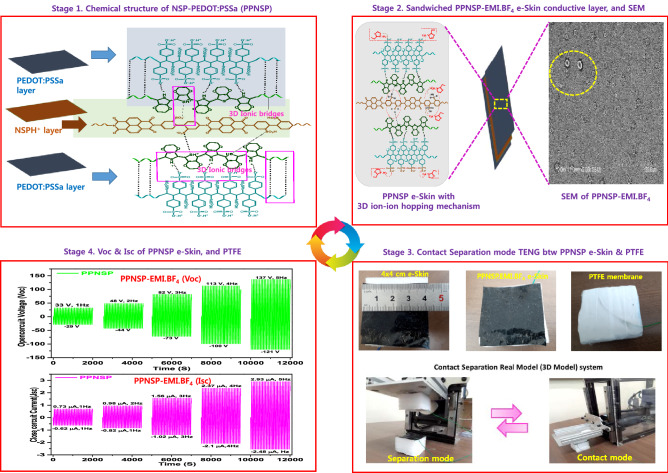
Schematic representation of PPNSP-EMI.BF_4_-PTFE TENG for harvesting energy through ionic electrets and ion-hopping mechanisms. Stage 1. Synthesis of NSP.H^+^s: PEDOT: PSSa composite film, Stage 2 Fabrication of ionic net-worked conductive composite electronic skin (e-Skin); Stage 3. Size of (4 cm × 4 cm) PPNSP.EMI.BF_4_ e-skin and PTFE, and vertical contact separation mode TENG; Stage 4. The Voc and Jsc of conductive PPNSP-EMI.BF_4_-PTFE e-Skin TENG.

Besides, the determination of the present work is to fabricate an ultrafast solvent drop-casting method to produce a combined networked electropositive ionic layer of PEDOT: PSSa—EMI.BF_4_-NSP (PPNSP) e-Skin strongly follows an ion-ion hopping mechanism (stages 1 and 2) using ionic electrets. The ionic conductivity, and ion exchange capacity of PPNSP are increased up to 3.3 times and 3.5 times through ionic electrets by an ion hopping mechanism that established that the higher density of excess protons (H^+^ ions) on the active surface can activate polarized charges to produce a higher TENG output voltage (Voc) and output currents (Isc) when interacting with the electronegative PTFE surface by contact separation mode (stages 3 and 4). The developed PPNSP-PTFE-TENG system is a virtuous candidate for the generation of higher Voc and Isc through a ion-ion hopping mechanism due to its significant benefits, such as a π–π stacked layer that helps to push and pull quick response to travel the ions via interconnected neural networked knots when undergoing contact-separation time. As a result, the novel PPNSP.EMI.BF_4_-PTFE TENG produced the Voc and Jsc values from 49.1 V and 1.03 µA at 1 Hz, 74 V and 1.45 µA at 2 Hz, 122.3 V and 2.23 µA at 3 Hz, and 171 V and 3.6 at 4 Hz, and 195.5 V and 4.43 µA at 5 Hz, respectively, 92 V and 2 mA, 74 V and 1.4 mA, 52 V and 0.93 mA, 32 V and 0.3 mA open-circuit voltages Voc and Isc at 5 Hz, 4 Hz, 3 Hz, 2 Hz, and 1 Hz, respectively, as shown in Fig. 1.

## Experimental

### Methods

The NSP.H^+^, PPNSP and PPNSP-EMI.BF_4_ e-skin were determined using FT-IR spectroscopy of IFS66 V/S & HYPERION 3000, Bruker Optiks (Germany). XRD of the membranes was measured using a DMAX-Ultima III XRD in the class of 5° to 60°. Tensile-Stress Curves and Thermogravimetric Analysis: The tensile strength, modulus properties and elongation of the films have been determined using a table-top universal testing machine (Autograph AGS-5 kN, Shimadzu Corp., Japan.) furnished by a 1 kN load cell with a test speed rate of 10 mm/min. The gauge length among the grips was 10 mm. All samples were cut into a regular rectangular shape. TGA measurements of the composite film membranes were completed on a thermogravimetric analyzer (TG209F3) from NETZSCH (Germany) finished temperatures ranging among 40 °C and 800 °C in N_2_ gas at a heating rate of 10 °C/min. All polymeric films were air dried, and SEM interpretations were carried out on an FEI Sirion FE-SEM, 30 kV microscope. The films were carefully dried before capturing the images, and the superficial morphology, and cross-sectional images were studied. The output signals of NSP.H^+^, PPNSP, PPNSP-EMI.BF_4_ e-skin, and PTFE were achieved by intermittently forced and free by income of an oscillator, and the power output was measured using Keithley Digital Multi Meter (KDMM). Consequently, all experiments were determined the impact force via load cell of YC33-5K (SETECH) at numerous frequencies of 1 Hz, to 5 Hz, correspondingly.

### Synthesis of NSP-H^+^ oligomers

The preparation of an NSP-H^+^ co-polyimide membrane with a ≈ 50% degree of sulfonation (DOS) was conducted. Initially, a 100 mL completely dried 2-neck flask was added to 2.0 g (5.8 mmol) of 4,4′-diamino-[1,1′-bphenyl]-2,2′-disulfonic acid (BDSA)], 20 mL of pure m-cresol, and 3.0 ml (3.6 mmol) of pure triethyl amine (TEA) successively under nitrogen flow with stirring at room temperature. After BDSA was dissolved, 3.116 g (11.6 mmol) of naphthalene dianhydride (NTDA), 1.16 g (5.8 mmol) of 4, =4′-oxydianiline (ODA), and 1.51 g (2.2 mmol) of phenyl formic acid have been added, sequentially. While agitation, the reaction mixture was reached to 80 °C and maintained for 4 h, and increased up to 190 °C for 20 h and cooled to 70 °C. An additional 25 mL of m-cresol was added for diluting the viscous liquid, and then poured into 300 mL of acetone. The polymeric fibers were separated by filtered, cleaned using acetone, and desiccated in vacuum. NSP.H^+^.TEA co-polyimide fibers were initially treated with 3 N HCl for 12 h to generate free sulfonic acid (NSP.H^+^), washed with DI water, and dried to obtain NSP.H^+^ oligomers^20^, (Supporting information).

### Synthesis of NSP^−^.H^+^ film from NSP^−^.H^+^ oligomers

NSP.H^+^ ionic film was prepared by solution casting methods. First, 1.0 g of NSP.H^+^ ionic fibers were dissolved in DMSO (15 mL), and stirred for 12 h to obtain a consistent solution, casted on a flat glass Petri dish, and vaporized at 90 °C for 24 h to obtain NSP.H^+^ film with a thickness of 75 µm^20^, (Supporting information).

### Fabrication of the PPNSP, and PPNSP-EMI.BF_4_ e-Skin from NSP.H^+^ film

**Figure 2:**
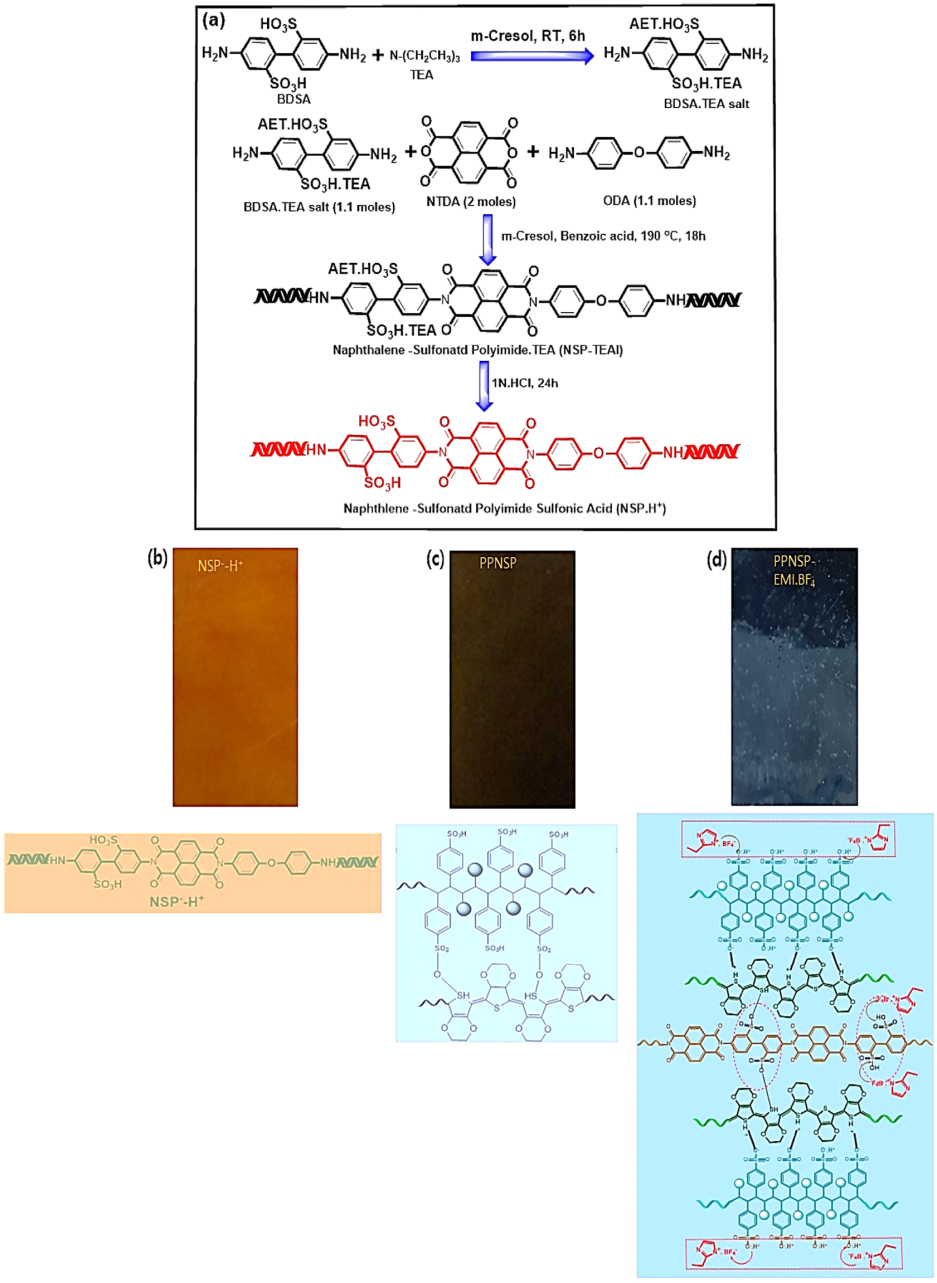
(**a**) Synthesis method of NSP.H^+^, and digital images and chemical structures of (**b**) NSP.H^+^, (**c**) conjugated conductive PEDOT: PSSa film; (**d**) ion hopping mechanism between PPNSP and EMI.BF_4_ with strong ionic-ionic interactions between host PPNSP and guest EMI.BF_4_ for generation of higher Voc and Jsc.

PPNSP composite film was fabricated by an ultrafast all-solution process. 10 wt% of DMSO was added into the aq. PEDOT: PSSa commercial solution at RT and stirred for 4 h to obtain a PEDOT: PSSa homogeneous solution. Next, the NSP.H^+^ film was submerged in a PEDOT: PSSa homogeneous solution for 12 h to generate a precast PPNSP composite membrane, and dried out at 70 °C for 5 h. Next, PPNSP e-Skin was immersed in 50% EMI.BF_4_ in DMSO for 12 h to generate PPNSP-EMI.BF_4_ composite e-skin for the TENG experiments, Fig. 2, (Supporting information).

### Fabrication of the contact-separation PPNSP-EMI.BF_4_-TENG

The fabrication and working principle of the contact-separation PPNSP e-skin TENGs are discussed. A methodical understanding of PPNSP TENGs has been designated in wide-ranging studies^18,28^. At this juncture, the assembly of the characteristic model was depicted in the Results and discussion part. First, NSP.H^+^ electrode was cut into sizes of 4 cm × 4 cm = 16 cm^2^, and attached on Al electrode layer. Then, the fabricated NSP.H^+^-Al conductor was closed to viable flexible foam to decrease the reflecting impact force while contact and separation is progressed. Then, a load cell was linked to the upper part of the Al conductor. A similar protocol was followed for the other two PPNSP-Al and PPNSP-EMI.BF_4_ -Al electrodes^39^.

Second, the Al electrode was positioned glued on the PTFE film at 4.0 cm × 4.0 cm along with polyurethane flexible foam. In the meantime, a linear oscillator is composed of a DC motor with eccentric arrangement steadily oscillated with a linear slider. The maximum oscillation amplitude was 40 mm. The upper portion of the PPNSP-EMI.BF_4_ e-skin was adjourned using a cantilever-style beam that was linked to the linear slider. The careful setup of the overall system give rise to in slight contact between the PPNSP-EMI.BF_4_ e-skin, and PTFE film, though the slider oscillation was steady to collect the current output data^40^.

## Results and discussion

### Preparation of naphthalene SPI (NSP.H^+^), NSP.H^+^-PEDOT: PSSa (PPNSP), PPNSP-EMI.BF_4_ composite e-skins

Figure 2a shows the synthesis of NSP^-^. H^+^ oligomer film by the sequential addition of monomers of BDSA. TEA, NTDA, and ODA in m-cresol using a literature method by random copolymerization through the addition of copolymers in a one-pot method. Next, NSP.H^+^. TEA was subjected to a proton exchange reaction in 3 N HCl to generate NSP^-^. H^+^ oligomer membrane, Fig. 2b. Next, the PPNSP film was fabricated using the solvent dip-coating method by mixing NSP.H^+^ oligomer film in 20 wt% PEDOT: PSSa in DMSO, Fig. 2c. Later, ionic networked PPNSP-EMI.BF_4_ composite film was synthesized using the dip-coating method by immersing the PPNSP film prepared in 20 wt% EMI.BF_4_ in DMSO for 12 h, respectively. During dip-coating process, EMI.BF_4_ is permeated into the host PPNSP through an ion hopping mechanism through a solvent sorption method. Additionally, long chain oligomers can show inter- and intramolecular charge transfer complex (CTC) singularities intercalated between SO_3_H groups with EMI.BF_4_. In addition, the hydrophilic SO_3_H functional groups were strongly intercalated with each other between PPNSP and EMI.BF_4_ through hydrophilic-hydrophilic interactions to generate higher Voc and Jsc^39,40^.

### Polymerization of PEDOT: PSSa, and interaction of PPNSP, and EMI.BF_4_

**Figure 3:**
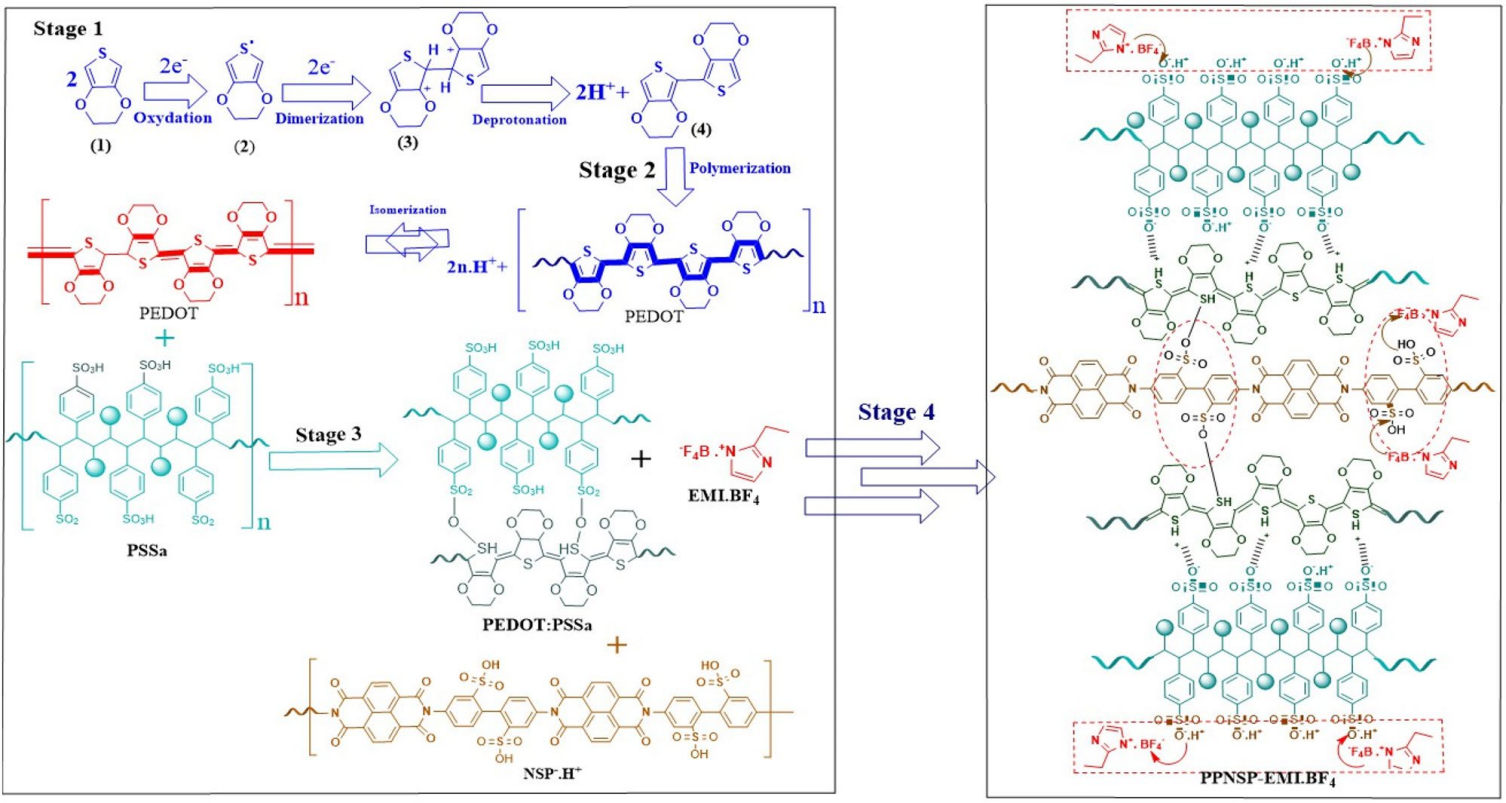
Mechanistic approach of the formation of oxidative and dimerization of EDOT, stage 1; polymerization and isomerization of EDOT, stage 2; Ionic interactions of EMI.BF_4_ with NSP^-^. H^+^, and PEDOT: PSSa, stage-3; and ion hopping mechanism between PPNSP and EMI.BF_4_, stage 4.

Figure 3 shows the mechanistic approach of the formation of PPNSP: EMI.BF_4_ e-skin TENG. From oxidation and dimerization of ethylene-dioxythiophene (EDOT) through an oxidative polymerization procedure catalyzed by metal cations, and the universal oxidant is present in various organic solvent systems. The stage 1, showed the oxidation of EDOT (1) monomers into unstable cationic radical structures (2). The unstable radicals were rearranged into a stable dimer (3) by reaction steps that involved combination and deprotonation, as shown in stage 2. In addition, a neutral PEDOT chain itself has a conjugated network with alternate double bonds with a low energy band gap and low oxidation potential. Ionic interactions were occurred with NSP.H^+^, and PEDOT: PSSa to generate the PPNSP e-skin, stage 3. Next, the PPNSP was soaked in 20% EMI.BF_4_ in DMSO to generate the PPNSP: EMI.BF_4_ composite e-skin, stage 4. Continuous recurrence of those steps resulted in the formation of well-dispersed, and doped PEDOT: PSSa solution showed the chemical structures of 3,4-ethylenedioxythiophene (EDOT), and poly(3,4-ethylenedioxythiophene) (PEDOT). The structure of the as-prepared PEDOT is made up of benzoid, and quinoid forms. The benzoid structure possesses a π-electron localized, conjugated structure that remains largely unaffected by external sources. In contrast, the quinoid form of PEDOT owns a delocalized state of π-electrons, which can be strongly exaggerated by solvent treatment^41^. In the electrically active, oxidized state, there remains a positive charge on every PEDOT polymer chain. The charges on the backbone were balanced with an anions are from small molecules or a macromolecular anions such as poly (4-styrene sulfonic acid) (PSSa). This higher charge transfer performance of the newly developed PPNSP: EMI.BF_4_ e-skin TENG through an ion-hopping mechanism that induces high ionic conductivity and tuned the mechanical properties, resulting from strong ionic interactions among the NSP.H^+^, EMI.BF_4_, and PEDOT: PSSa, and interconnected networked polymer matrix^42,43^.

### SEM analysis of NSP.H^+^, PPNSP, and PPNSP-EMI.BF_4_ composite films

**Figure 4:**
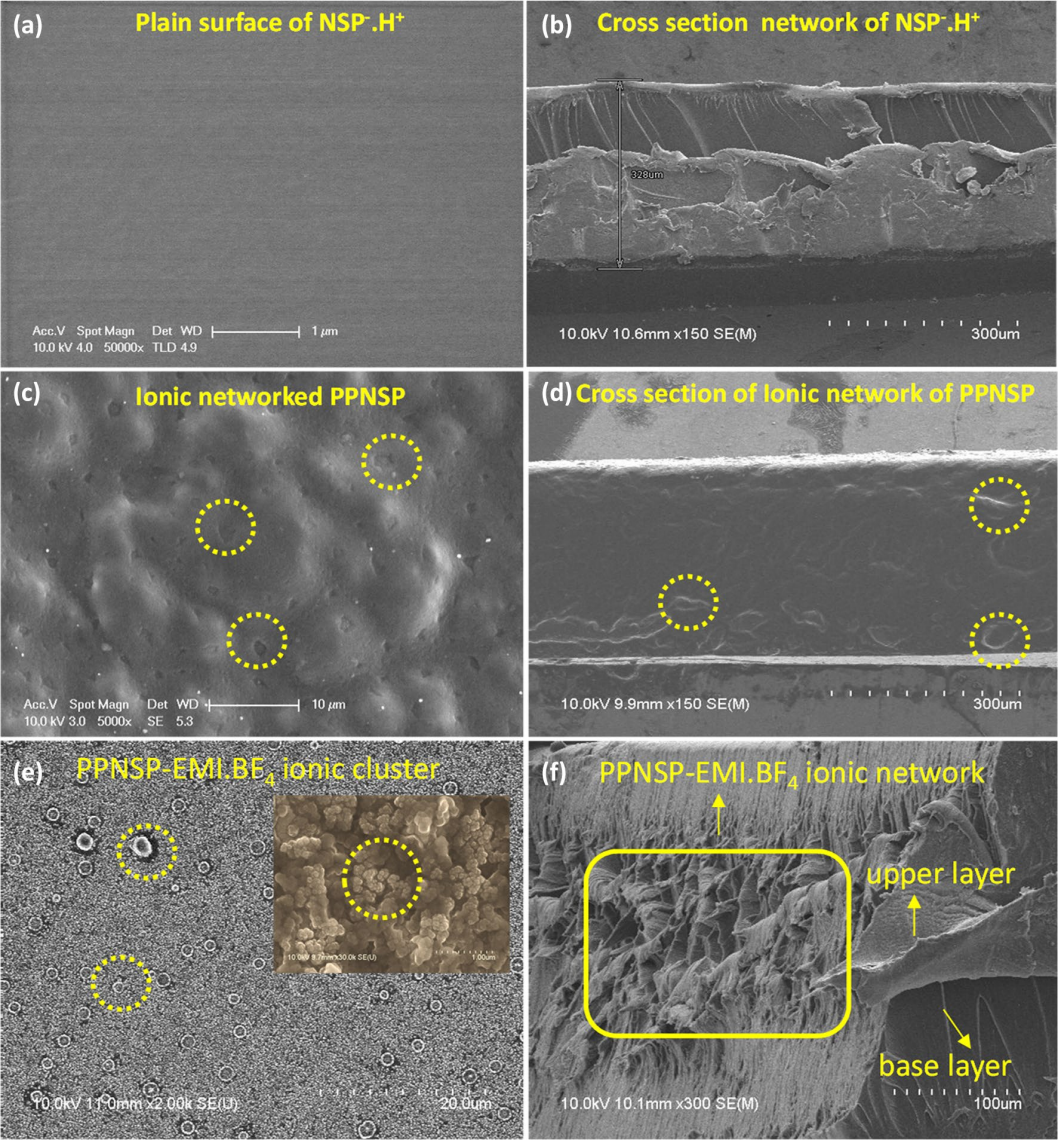
FE-SEM images of the surface morphologies of NSP.H^+^, PPNSP, and PPNSP-EMI.BF_4_ composite films; (**a**, **b**) regular plain surface; ionic networked submicron to micron hallow grooves and knots on the surface, (**c**, **d**); ionic clusters on the surface of PPNSP film by aggregations of EMI.BF_4,_ (inset image, strongly glued PPNSP-EMI.BF_4_ ionic clusters, which can help to promote the ion hopping mechanism, at 1 μm magnification) (**e**), and loosely bonded ionic networks in the cross-sectional view of PPNSP-EMI.BF_4_ composite film (**f**).

Scanning Electron Microscopy (SEM) images of the NSP.H^+^ and PPNSP films are shown in Fig. 4 and reveal the surface morphologies. In particular, Fig. 4a and b show the wrinkle-free plain surface with no obvious changes. However, the inbuilt hydrophilic –SO_3_H groups were attached to the host polyimide co-blocks that could intercalate and generate nano level distances between the oligomeric networked chains. Significant submicron-sized porous grooves and micron-sized crests with tightly packed networks were found on the surface, as shown in Fig. 4c. The hollow groves and crests were created due to a significant ionic network on the surface morphology. In the cross-sectional view, hydrophilic and hydrophilic interactions take place between NSP.H^+^, and PEDOT: PSSa through hydrogen bonding that created a loosely bound network between them, Fig. 4d ^21^. The hydrophilic-hydrophilic ionic system can establish a flexible interpenetration complex between NSP^–^H^+^, and PEDOT: PSSa to improve the charge densities on the polymeric surface. The ionic networked morphology can exchange ions through strong ionic knots, which can travel through hydrophilic ionic channels and strongly support the ion hopping mechanism. After impregnation of EMI.BF_4_ IL, the thickness of PPNSP e-Skin was increased due to swelling of ionic liquid. Figure 4e shows the strong spherical aggregations on the surface by ionic clusters^44^. In addition, the inset image clearly indicates the ionic clusters at nanoscale levels. In the cross-sectional view, a loosely bounded net-like channeled network appears due to the strong intercalation between PEPDOT: PSSa and EMI.BF_4_ IL. Additionally, the high-resolution surface and cross-sectional images display the formation of zigzag net-like channels. In particular, EMI^+^ cations were deposited on the hydrophilic regions as bright spots, which clearly indicate the formation of PPNSP-EMI^+^ by an ion hopping mechanism, as shown in Fig. 4f. The homogeneity of the blend membrane confirmed the strong ionic interactions, which included ionic cross-linking and hydrogen bonding between EMI.BF_4_ and the PPNSP composite membrane, resulting in the enhanced interfacial compatibility and mechanical stiffness of the composite membrane. In addition, ionic cross-linking and hydrogen bonding can generate higher charges on the surface. These charges can enhance the open circuit voltage and short circuit currents. The overall morphologies were strongly justified to accelerate the charges within the charge transfer complexes when a contact-separation mode TENG was performed^39^. The FT-IR, XRD, Stress–strain (SS) curves, and TGA analyses of NSP.H^+^, PPNSP, and PPNSP.EMI.BF_4_ were reported in Supporting information.

## Set-up of PPNSP-EMI.BF_4_ e-skin TENG for generation of voltages and currents

**Figure 5:**
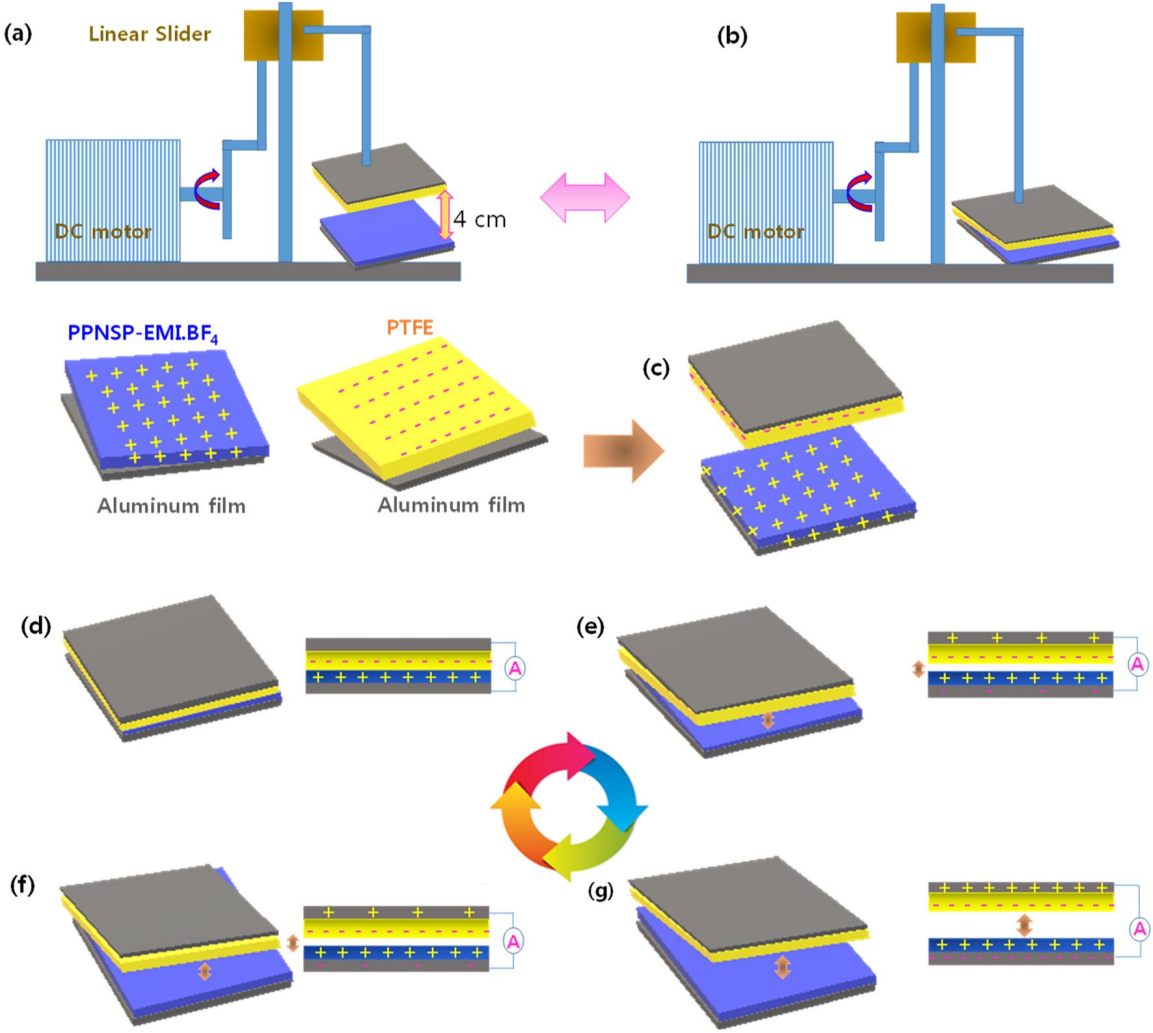
Mechanical operation of PPNSP-EMI.BF_4_ e-skin TENG at (**a**) separation state, (**b**) fully contacted state (**c**) initial condition, (**d**) full contacted, (**e**) separated, (**f**) fully separated condition and (**g**) contacted again of TENG results of NSP.H^+^, PPNSP, and PPNSP-EMI.BF_4_ e-Skin.

Figure 5a and b shows the mechanical setup of the PPNSP-EMI.BF_4_ e-skin TENG in full separation and contact mode of state, periodically, to generate the actual triboelectric current. The energy harvesting principle strictly follows the contact electrification approach by the contact and separation of PPNSP-EMI.BF_4_ and PTFE under various applied frequencies. The working principle of the proposed PPNSP-EMI.BF_4_ e-skin TENG was explained by the PPNSP surface, and the aluminum (Al) electrodes were initially free of charges, as shown in Fig. 5c. When the oscillation was underway by the linear slider by the DC motor, the triboelectrically negative PTFE was in superficial contact on the surface of PPNSP-EMI.BF_4_. The PTFE surface was converted to temporarily negatively charged, and in contrast, PPNSP-EMI.BF_4_ turned positive owing to the contact electrification process, Fig. 5d. During the contact electrification process, the generated electrical charges are retained on the surface for a longer time due to the insulating properties of the surface. The PTFE surface showed superior charge separation when it was separated from PPNSP-EMI.BF_4_ e-skin. At this time, the Al electrodes were strongly persuaded to collect charges by PPNSP-EMI.BF_4_ e-skin and PTFE surfaces remained neutral, the top electrode was positively charged, and the bottom electrode was negatively charged^45^. During the separation process, the generated charges were transformed through an external load, and current flow occurred, Fig. 5e. When the two electrodes were completely separated, the current was clogged and reached the equilibrium state, Fig. 5f. When both the surfaces of PPNSP-EMI.BF_4_ e-Skin and PTFE were locked together, and the electrostatic induction was high and broke the equilibrium state, resulting in charge redistribution of Al electrodes via acceptance and release of ions, as shown in Fig. 5g. As a result, the current flows in the reverse direction. Till the two polymeric layers were fully contacted, the charge-transfer process disappeared, and no charges were generated at all^38,39^.

### TENG result of NSP.H^+^-PTFE, PPNSP-PTFE, and PPNSP-EMI.BF_4_ e-skin-PTFE

**Figure 6:**
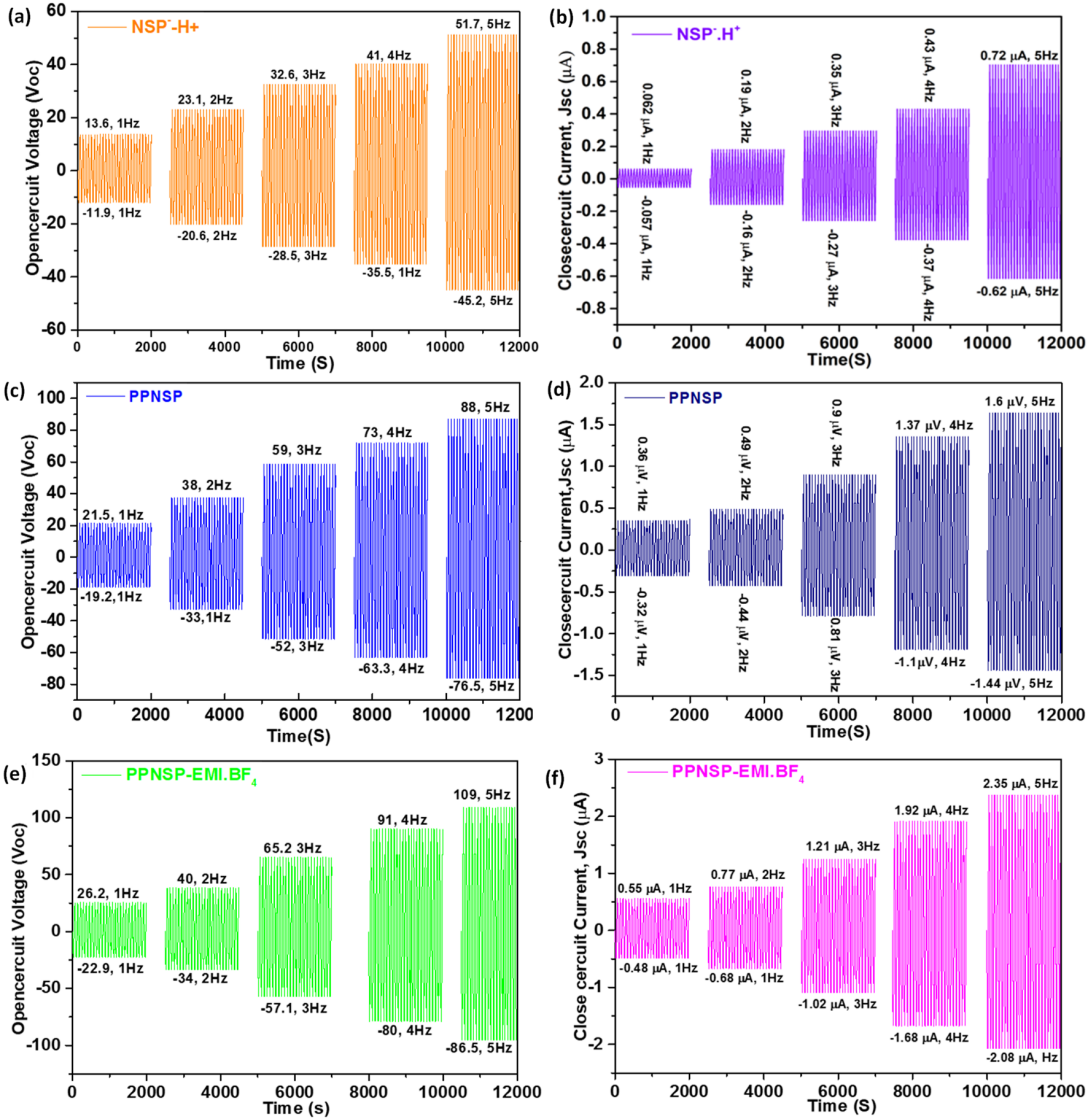
The output voltages (Voc) and close circuit currents (Isc) under numerous feedback conditions in open circuit structures. The Voc and Isc of (**a**, **b**) NSP.H^+^-TENG; (**c**, **d**) PPNSP-TENG; and (**e**, **f**) PPNSP-EMI.BF_4_ e-skin-TENG.

To authenticate the TENG principle, we first investigated the electric output of the NSP.H^+^ against the PTFE film through the contact separation mode. The open-circuit Voc of the NSP.H^+^ at 1 Hz surged from − 11.6 to 13.6 V upon separated. Additionally, the charge transferred between the Al electrodes instantaneously generated a short-circuit current Jsc. The peak value of Jsc reached from − 0.057 to 0.062 µA, as shown in Fig. 6a and b. By gradually changing the speed of the linear motor from 1 to 5 Hz, different Voc and Jsc values were observed. The generated Voc and Jsc were achieved from − 20.6 to + 23.1 V, − 0.0.26 to 0.26 µA at 2 Hz, from − 28.5 to 32.6 V and from − 0.27 to 0.35 µA at 3 Hz, and from − 35.5 to 41 V and from − 3.7 to 0.43 µA at 4 Hz and − 45.2 to 51.7 V and − 0.62 to 0.72 µA at 5 Hz, respectively. Next, the Voc and Jsc of the PPNSP TENG showed an output voltage of − 19.2 V to 21.5 V, and from − 0.32 to 0.36 µA at 1 Hz, from − 33 to 38 V and from − 0.44 to 0.49 µA at 4 Hz, from − 52 to 59 V, and from − 0.81 to 0.9 µA at 3 Hz, from − 63.3 to 73 V, and from − 1.1 to 1.37 µA at 6 Hz, − 76.5 to 88 V and − 1.44 to 1.6 µA, respectively, as shown in Fig. 6c and d. Significant enhancements were observed in both voltage and current after doping with EMI.BF4 IL into the PPNSP film by dip-coating method^45^. Significant enhancement was observed in both voltage and current after doping with EMI.BF_4_ IL into the PPNSP film by dip coating method. The homogeneous deposition EMI.BF_4_ into PPNSP, quantum jump enhancement was observed, and the quantitative output performance of PPNSP-EMI.BF_4_ was determined due to active mobility of EMI.BF_4_ ions within the host PPNSP polymer film as well as the surface. The PPNSP-EMI.BF_4_-PTFE TENG provided Voc and Jsc values from − 22.9 to 26.2 V and from − 0.48 to 0.55 µA at 1 Hz, from − 34 to 40 V and from − 0.68 to 0.77 µA at 2 Hz, from − 57.1 to 65.2 V and from − 1.02 to 1.21.08 µA at 3 Hz and from − 80 to 91 V and from − 1.68 to 1.92 µA at 4 Hz and − 86.5 to 109 V and − 2.08 to 2.35 µA at 5 Hz, respectively, as shown in Fig. 6e and f. In specific, it can be seen that the PPNSP-EMI.BF_4_-PTFE TENG showed higher electrical output than the other two systems (i.e., NSP.H^+^-PTFE, and PPNSP-PTFE TENG) owing to the availability of abundant mobile BF_4_^−^ ions from EMI.BF_4_ IL. The present result have been justified the concept by the Jang et al. have displayed higher conductivity through our PPNSP-EMI.BF_4_ e-skin TENG in comparison with NSP.H^+^ and PPNSP TENGs due to the addition of EMI.BF_4_^46^.

### Performance characteristics of PPNSP.EMI.BF_4_-PTFE TENG

**Figure 7:**
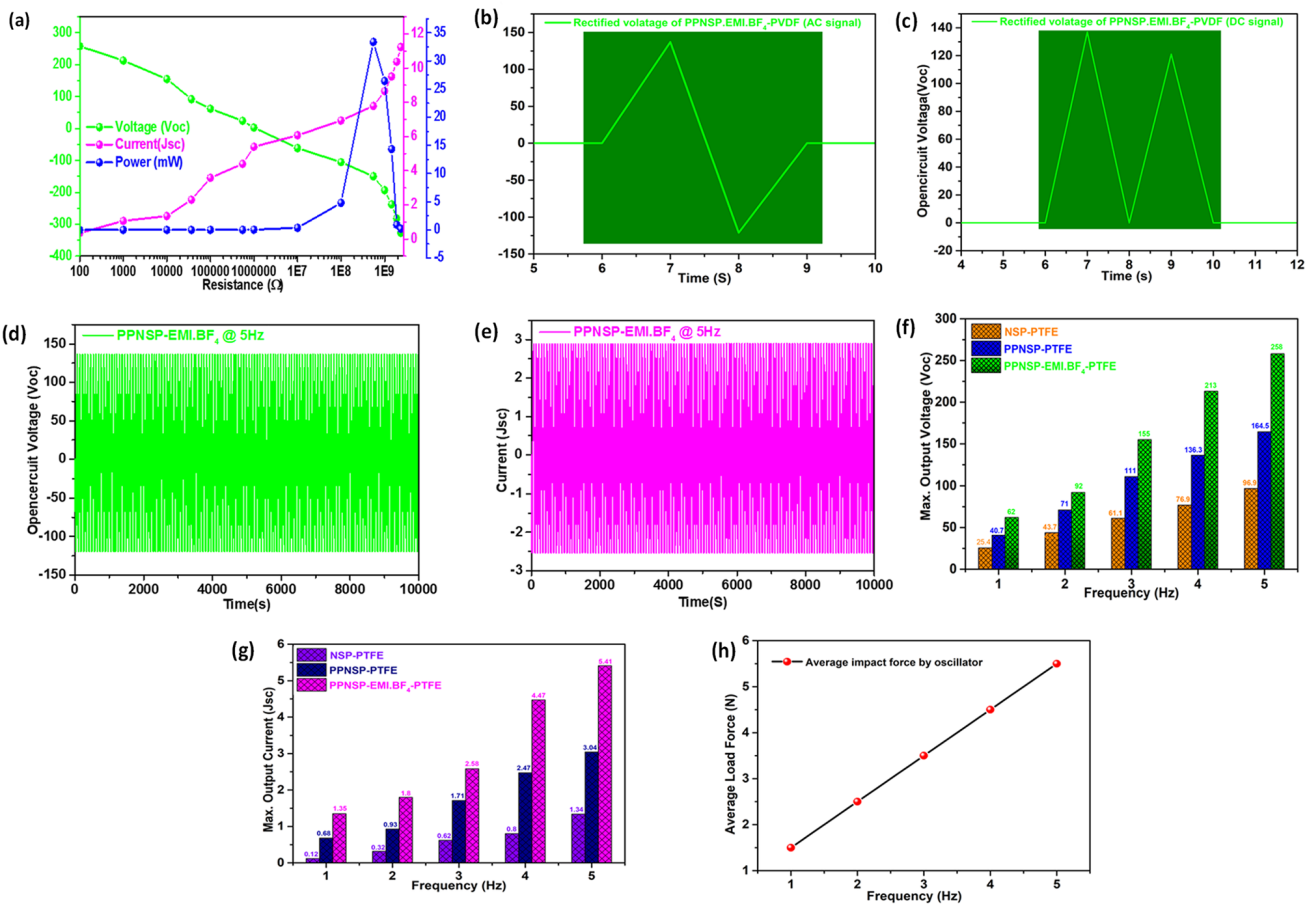
(**a**) Load resistance analysis and power density calculations such as open circuit voltage (Voc), short circuits current (Jsc), and power output, (mW) against external load resistances (Ω) at 5 Hz; (**b**) rectified voltage of AC signal, (**c**) DC signal; (**d** and **e**), stability and durability performance tests of Voc and Jsc of 10,000 cycles at 5 Hz. (**f**) Voc, (**g**) Jsc, and (**h**) Impact forces of PPNSP.EMI.BF_4_ e-skin TENG at various applied frequencies.

Additional experiments of load resistance analysis, power densities, and device constancy, were carried out using the PPNSP.EMI.BF_4_ e-skin-PTFE TENG at 5 Hz applied frequency, as shown in Fig. 7. The peak instantaneous Voc and Isc of PPNSP.EMI.BF_4_ e-skin-PTFE TENG were measured at 5 Hz oscillation. It was noted that the peak Voc decreased with increasing resistance, whereas Jsc increased. Consequently, the device output increased when the load resistance was increased from 100 ohm Ω to 570 MΩ. The areal power density of the proposed device at a 5 Hz applied load was evaluated using the following equation.

$$ {\text{P}}_{{\text{A}}} = {\text{V}}^{2}/({\text{R}} \times {\text{A}}) $$

where V is the generated output voltage from PPNSP.EMI.BF_4_-PTFE TENG device, R is the load resistance, and A is the device area (A = 4 cm × 4 cm = 16 cm^2^). The areal power density of the device was 33.2 mW, and the load resistance was 570 MΩ. This indicated that 570 MΩ was the load-matching resistance for achieving the maximum output power required for real-time applications, as shown in Fig. 7a. The unidirectional output can be stored in energy storage systems such as capacitors, and batteries and the rectified Voc of PPNSP.EMI.BF_4_-PTFE TENG device is shown in Fig. 7b and c. The performance stability of Voc and Jsc of PPNSP.EMI.BF_4_-PTFE TENG system was checked at a 5 Hz applied frequency, and it was stable for ~ 10,000 cycles without any fluctuations, as shown in Fig. 7d and e. The durability and stability of the proposed system showed exceptional harvesting properties and superior mechanical strength.

The performance of Voc, and Jsc of NSP.H^+^-PTFE-TENG > PPNSP-PTFE-TENG > PPNSP-EMI.BF_4_ e-skin-PTFE-TENGs. The e-skin-TENG gradually increased with increasing ionic density both inside the network and outside the surface. Next, when the impact force was increased by the oscilloscope, surface charges were generated through the ion hopping induction mechanism and surged the higher Voc, and Jsc values. The output Voc, and Jsc of PPNSP.EMI.BF_4_-TENGs produced 316% and 300% at 5 Hz, respectively, as shown in Fig. 7f and g. The load cell YC33-5 K (SETECH) was utilized to measure the contact force, and the results are shown in Fig. 7h. Based on the obtained research data, the impact force was gradually increased with respect to the applied contact frequencies. When the impact force is high, an additional effective area is induced, and subsequently, higher output voltage and currents are generated. The Voc values of PPNSP.EMI.BF_4_-TENGs are 1.6, and 2.6 times higher than that of PPNSP-PTFE-TENG and NSP-PTFE-TENG at 5 Hz, and the Jsc values of PPNSP.EMI.BF_4_-TENGs are 1.8 and 4 times higher than that of PPNSP-PTFE-TENG, and NSP-PTFE-TENG at 5 Hz, respectively, due to strong ionic interactions occurring through an ion hopping mechanism to transport ions. Additionally, a mechanistic explanation is suggested when EMI.BF_4_ IL interacts with the ionic sites of NSP.H^+^, and PPSNP polymers and exchanges ions. The Voc, and Isc of PPNSP. EMI.BF_4_-TENGs showed superior triboelectric properties compared to PPNSP-PTFE TENGs and NSP.H^+^ TENGs owing to the availability of large ionic clusters by invasion of EMI.BF_4_ IL within the networked channels inside and on the surface of the polymer network. Therefore, we modified the NSP.H^+^, PPSNP, and PPNSP. EMI.BF_4_ composite surfaces for generating high power output through doping of EMI.BF_4_ into the NSP.H^+^, and subsequently, dip coating of the PEDOT: PSSa conducting polymer to generate higher ionic conductivities. Next, we conducted experiments to regulate the impact force at various applied frequencies.

## Conclusion

In this research, we have demonstrated an ionic networked polymer TENG based on PPNSP.EMI.BF_4_-PTFE with ion-ion hopping alternates hydrophilic nano-channels integrated with sulfonic acid groups, resulting in superior electro-chemo-mechanical properties. Well-arranged hydrophilic sulfonic acid groups have good compatibility with both PEDOT: PSSa, and EMI.BF_4_ to penetrate at atom–atom interactions owing to PPNSP.EMI.BF_4_ composite film thereby provided constant interconnected ion-ion interactions to transport charges through ionic nano-channels to enhance Voc, and Jsc performance and robustness. The FE-SEM analysis clearly revealed the ion-ion nano-channels within the polymer network that trigger the charges very fast when PPNSP.EMI.BF_4_ e-skin interacted with the PTFE surface. The SS curves of the NSP.H^+^, PPNSP, and PPNSP.EMI.BF_4_ noticed the strong flexibility and resilience of the membranes to be compatible with the contact-separation mode TENG without damaging the membranes even after several cycles of contact-separation mode TENGs. Mainly, the PPNSP.EMI.BF_4_ e-skin displayed a dramatic increase in the tensile modulus, up to 131%, tensile strength, up to 174%, and elongation at break values up to 277% compared to those of its starting membranes, such as the NSP.H^+^ and PPNSP membranes. These synergistic effects were beneficial after incorporation of EMI.BF_4_ IL in the PPNSP membrane in designing a high-performance ion-mediated e-skin-based TENG that shows large affective charge induction to provide higher Voc, and Jsc. In particular, the NSP-H^+^ membrane was sandwiched by the PEDOT: PSSa conducting polymer, which enhanced superior conductivities.

The Voc and Jsc of the NSP.H^+^ at 5 Hz surged upto − 45.2 to 51.7 V and − 0.62 µA to 0.72 µA, respectively. Next, the PPNSP TENG showed an output voltage of at 5 Hz, − 76.5 V to 88 V and − 1.44 to 1.6 µA, respectively. On the other hand, PPNSP-EMI.BF_4_ e-skin was shown upto − 86.5 V to 109 V and − 2.08 to 2.35 µA at 5 Hz owing to EMI.BF_4_ IL interpenetrated within the polymer network, which enhanced the accumulation of high power densities. Significant enhancement was observed in both voltage and current after doping with EMI.BF_4_ IL into the PPNSP film by dip coating method. The homogeneous deposition EMI.BF_4_ into PPNSP, quantum jump enhancement was observed, and the quantitative output performance of PPNSP-EMI.BF_4_ was determined due to active mobility of EMI.BF_4_ ions within the host PPNSP polymer film as well as the surface. In specific, it can be seen that the PPNSP-EMI.BF_4_-PTFE TENG showed higher electrical output than the other two systems (i.e., NSP.H^+^-PTFE, and PPNSP-PTFE TENG) owing to the availability of abundant mobile BF_4_^−^ ions from EMI.BF_4_ IL. Next, performance characteristics of PPNSP.EMI.BF_4_-PTFE TENG showed the load resistance analysis, power density calculations, and device stability at 5 Hz applied frequency, and load resistance was increased from 100 ohm Ω to 570 MΩ. The areal power density of the device was 33.2 mW, and the load resistance was 570 MΩ. Additionally, the unidirectional output was stored in energy storage systems such as capacitors and batteries and the rectified Voc of PPNSP.EMI.BF_4_-PTFE e-skin TENG device. The performance stability of Voc and Jsc of PPNSP.EMI.BF_4_-PTFE TENG system was checked at a 5 Hz applied frequency, and it was stable for ~ 10,000 cycles without any fluctuations. The durability and stability of the proposed system showed excellent harvesting performance and superior mechanical strength without any surface damage. The present results have suggested that the controlled self-assembly process for strong ion-ion connections and ion transport nanochannels can be used for tailoring superior TENG applications, which are potentially required for next-generation electronic products such as wearable soft electronics, flexible displays, and smart mobile phones.

## Supplementary Information

Supplementary Information.
