# Supplementary material for: Integrated electronic skin (e-skin) for harvesting of TENG energy through push–pull ionic electrets and ion-ion hopping mechanism
Source: Sci Rep. 2022 Mar 9;12:3879. doi: 10.1038/s41598-021-04555-3 (PMC8907315; doi:10.1038/s41598-021-04555-3)
Supplement: Supplementary file 1 — Supplementary Information. [file 41598_2021_4555_MOESM1_ESM.docx]

**Supporting Information**

**Integrated Electronic Skin (e-Skin) for Harvesting of TENG Energy through Push-Pull Ionic Electrets and Ion-ion Hopping Mechanism**

Ravi Kumar Cheedarala*^1^ and Jung Il Song*^1^

^1^The Research Institute of Mechatronics, Department of Mechanical Engineering, Changwon National University, Changwon city, S. Korea.

Corresponding Authors Emails: [ravi@chagwon.ac.kr](mailto:ravi@chagwon.ac.kr) and [jisong@changwon.ac.kr](mailto:jisong@changwon.ac.kr)

**SI.1. Preparation of NSP.H+, PPNSP and PPNSP.EMI.BF_4_**

**Fig. SI-1**. Fabrication method of PPNSP-EMI. BF_4_ composite film **for harvesting energy through 3D ionic electrets, and an ion-hopping mechanism. Step 1. Casting of NSP.H^+^ film from NSP.H^+^ Oligomer solution. Step.2 Preparation of PPNSP e-skin by dip-coating of PEDOT:PSSa on NSP.H^+^ film. Step.3. Absorption of 20 V% of EMI. BF_4_ IL in DMSO within PPNSP film to generate PPNSP.EMI.BF_4_ e-skin.**

**SI.2. FT-IR, XRD, Stress-strain (SS) curves,** and **TGA analyses of NSP.H+, PPNSP and PPNSP.EMI.BF_4_**

Figure SI-2. FT-IR (a); XRD (b); SS curves (c); and TGA curves (d) of NSP-.-H^+^, PPNSP, and PPNSP-EMI. BF_4_ composite e-skins

**FT-IR**

FT-IR spectra are quite helpful to identify structural and bonding changes in the synthesized novel NSP-based ionic 3D electret membranes. **Figure SI-2a** shows the FT-IR spectra of NSP.H^+^, PPNSP, and PPNSP-EMI. BF_4_ e-skins. The characteristic absorption peaks of NSP.H^+^ were shown at 628 cm^−1^ (-SO_2_), 770 cm^−1^ (-SO_2_), 880 cm^−1^ (-SO_2_), 1032 cm^−1^ (SNS), 1196 cm^−1^ (ν sym, O=S=O), 1250 (ν, C-N-C), 1344 cm^−1^ (ν, CNC), 1453 cm^−1^ (-OH, ben), 1505 cm^−1^ (-CN), 1554 cm^−1^(ν, -C=C), 1674 cm^−1^ (ν asym, -C=O), 1712 cm^−1^ (ν sym, -C=O), belong to amide-I, and amide-II, which correspond to the sulfonic acids on the oligomer chains of the NSP polymer. The FT-IR signals of PPNSP showed its typical signatures. For instance, the peaks at 622 cm^−1^ (-SO_2_), 695 cm^−1^ (-SO_2_), 723 cm^−1^ (SO), 770 cm^−1^ (-SO_2_), 830 cm^−1^ (CSC and SO), 921 cm^−1^ (CSC), 1082, cm^−1^ (OCO), 1137 cm^−1^ (C=C), 1193 cm^−1^ (OCO and SO_2_), 1451 cm^−1^ (OH, ben), 1498 cm^−1^ (-CN), respectively. The strong stretching bands at 1554 cm^−1^ (v, C=C), 1676 cm^−1^ (ν asym, -NH-C=O), and 1715 cm^−1^ (ν sym, -NH-C=O), belonging to amide-I, and amide-II, respectively, were moved to two units due to strong intercalation where ionic-ionic interactions occur between NSP-. H^+^ and PPNSP membranes. Next, the FT-IR spectrum of the PPNSP-EMI. The BF_4_ 3D ionic electret membrane is consistent with the chemical structure, which is composed of PPNSP and 1-ethyl-3-methylimidazolium tetrafluoro borate (EMI. BF_4_). The EMI. BF_4_ shows characteristic bands of C-H stretching in the imidazole ring stretching peaks at 1445 cm^−1^ and 1564 cm^−1^, imidazole H-C-C, and H-C-N twisting peaks at 925 cm^−1^ and 1191 cm^−1^, in-plane imidazole ring twisting at 828 cm^−1^, out-of-plane C-H bending of the imidazole ring at 738 cm^−1^, and imidazole C-C bending at 598 cm^−1^, respectively. The stretching peaks appeared at 294 cm^−1^, 1191 cm^−1^, 1121 cm^−1^, 1030 cm^−1^, 921, cm^−1^, and 830 cm^−1^[19].

The strong stretching signals of the BF_4_ group appear at 1191 cm^−1^ with a shoulder at 1230 cm^−1^, and the deformation signals at 738 cm^−1^ and at 658 cm^−1^ strongly support the presence of an imidazolium ring. Additionally, additional strong peaks appearing at 1121 cm^−1^ for SO_2_ and 1344 cm^−1^ with a left shoulder at 1329 cm^−1^ corresponding to the C=N of the imidazolium cation strongly support the presence of EMI. BF_4_ in the PPNSP-EMI. BF_4_ e-skin. As suggested in **Figure 2**, ionic clusters are formed by an ultra-ionic exchange reaction between the PPNSP membranes with EMI. BF_4_ is well-supported by the FT-IR studies [21]. In addition, 3D ionic electrets were developed during slow evaporation of DMSO from hydrophilic PPNSP, and EMI. BF_4_. Good dispersion of hydrophilic hydrophobic EMI. BF_4_ with the core PPNSP polymer enhances interfacial gluing by hydrophilic ionic centers and hydrophilic-hydrophilic ionic interactions and the formation of homogeneous PPNSP.EMI. BF_4_ e-skin can display higher ionic exchangeability and superior mechanical properties and performs as an ultrafast ion-exchange TENG electrode. The formation of homogeneous and hydrophilic-hydrophilic ionic interactions between NSP. H^+^, PPNSP, and PPNSP.EMI. BF_4,_ through both hydrogen and ionic binding, can display the highest power output through a 3D ionic electret hopping mechanism.

**XRD**

XRD patterns of the ionic 3D electrets networked NSP^-^.-H^+^, PPNSP, and PPNSP-EMI. BF_4_ e-skin were depicted in **Figure SI-2b**. Broad hallow humps at 18.3 and 23.6 2θ° was found to be amorphous owing to the formation of semi-crystalline components within the polymer network. However, it contains ionic 3D electrets that loosely connect although the ionic bonds between ions and molecules. The XRD pattern of the PPNSP membranes showed a sharp broad peak with superimposition of short crystalline peaks, which indicates strong crystallinity, and the peak appeared at 21.6 θ°. We investigated the XRD pattern of the PPNSP-EMI. BF_4_ ionic 3D electret membrane compared with those of the NSP.H^+^ and PPNSP membranes. The EMI. BF_4_ displayed a plasticizing effect and chelated strongly with both NSP.H^+^ and PPNSP to enhance the semi-crystallinity of the membranes. The NSP.H^+^ and PPNSP membranes do not show any phase separation due to the formation of strong intercalation through hydrophilicity by ionic-ionic interactions[19, 20] distinct crystalline peaks were at 12.5 and 26.5, 2θ°, showing strong evidence of stretchy crystallinity due to flexible ion-ion interactions within the membrane as a free-flowing network. In addition, the EMI. BF_4_ is strongly aggregated on the surface of the PPNSP due to the formation of ionic clusters. The broad peaks that were observed in the NSP.H^+^ and PPNSP membranes transformed into sharp peaks at 12.5 and 26.5 2θ°, and the degree of crystallinity is increased due to the establishment of ionic 3D electrets, bridges between NSP.H^+^, and PPNSP through EMI. BF_4_.

**Stress-strain (SS) Curves**

With the good deposition of PEDOT: PSSa on top of NSP.H^+^ base polymer through strong ionic bonding and reinforcement that can be expected. To demonstrate this reinforcement, tensile tests were performed for 3D ionic electrets, networked PPNSP, and PPNSP-EMI. BF_4_ e-skin [20]. Typical stress-strain curves are shown in **Figure SI-2c**, and their mechanical properties are compared in **Table 1**. The NSP. H^+^ showed weak behavior and softer behavior and displayed enhanced strain with less strength than PPNSP and PPNSP-EMI. BF_4_ membranes. The tensile strength of the NSP. H^+^ polymer matrix was showed a lower tensile strength at 25 MPa, and the strain was 16.6%. The novel PPNSP, and PPNSP.EMI. BF_4_ membranes showed enhanced tensile strengths of 32 MPa, and 40.2 MPa and strains were reduced gradually from 10.6 and 8, respectively. The tensile modulus is gradually increased from 1.5 GPa for NSP. H^+^, to 3.0 GPa, to 5.0 GPa for PPNSP.EMI. BF_4_, respectively. The tensile modulus was increased up to 400% from NSP.H^+^ membrane to PPNSP.EMI. BF_4_ e-skin by the strong gluing between the membranes through special and interionic interactions between the membranes. Moreover, EMI. BF_4_ can penetrate the whole polymeric network through interfacial ionic interactions within NSP and PPNSP to increase the movement of ions between membranes.

**Table 1.** Stress-strain (SS) curves and elongation at breaks of all the films**.**

| **Films** | **Tensile modulus**  **(GPa)** | **Tensile Strength**  **(MPa)** | **Elongation at Break (%)** |
| --- | --- | --- | --- |
| NSP.H+ | 1.5 | 25 | 16.6 |
| PPNSP | 3.0 | 32 | 10.6 |
| PPNSP.EMI.BF_4_ e-skin | 5.0 | 40.2 | 8 |

**TGA**

Well gluing effect between both PEDOT: PSSa, NSP.H^+^, and EMI. BF_4_ within the polymer matrix showed superior thermal properties. **Figure SI-2d** showed the TGA thermogram of NSP.H^+^, PPNSP, and PPNSP-EMI. BF_4_ e-skin were differ from each other. They displayed three-stage decomposition; the first stage involved the loss of SO_2_ molecules; the second stage involved the decomposition of aromatic polymer chains; and the third stage included the vaporization and elimination of volatile fragments. With the first stage between 50 °C and 295 °C, almost a 2-6% loss of the initial weight occurs in the case of PPNSP-EMI. BF_4_ e-skin, the weight loss is just 1% due to the strong plasticizing effect that occurs with EMI. BF_4_ [21]. The second decomposition stage of the NSP.H^+^ membrane showed up to 25%, and the other two membranes decomposed up to 60% weight loss at 445 °C to 450 °C, which involved decay of aromatic ring systems of the polymer. The third stage of decomposition, which ends at approximately 800 °C, involves a weight loss of approximately 20% from the carbon chains, which are decomposed into lightweight gases of CO_2_, CO, and NH_3_ and O_2_ gases. The thermogram of the NSP.H^+^ membrane differs from that of PPNSP and PPNSP-EMI. BF_4_ e-skin. This change might render due to PPNSP, and PPNSP-EMI. BF_4_ e-skin were hydrophilic after EMI. BF_4_ doping, and destroyed the crystalline structures. Eventually, after mixing with EMI. BF_4_, the structure of the PPNSP becomes irregular, with deterioration of the thermal properties.
